# Ocular surface microbiome in diabetes mellitus

**DOI:** 10.1038/s41598-022-25722-0

**Published:** 2022-12-13

**Authors:** Orathai Suwajanakorn, Vilavun Puangsricharern, Thanachaporn Kittipibul, Tanittha Chatsuwan

**Affiliations:** 1 Cornea and Refractive Surgery Unit, Department of Ophthalmology, Faculty of Medicine, Chulalongkorn University, Bangkok, Thailand; 2 Excellence Center of Cornea and Limbal Stem Cell Transplantation, Department of Ophthalmology, King Chulalongkorn Memorial Hospital, Bangkok, Thailand; 3 Department of Microbiology, Faculty of Medicine, Chulalongkorn University, Bangkok, Thailand; 4 Center of Excellence in Antimicrobial Resistance and Stewardship, Faculty of Medicine, Chulalongkorn University, Bangkok, Thailand

**Keywords:** Microbiology, Medical research

## Abstract

This cross-sectional, age- and gender-matched study included 20 eyes of non-diabetic subjects (non-DM group) and 60 eyes of type 2 diabetes mellitus (DM group). Subgroups of DM were classified by diabetic retinopathy (DR) staging into no DR (DM-no DR), non-proliferative DR (DM-NPDR), proliferative DR (DM-PDR), and by glycemic control (well-controlled DM; HbA1c < 7%, poorly controlled DM; HbA1c ≥ 7%). Conjunctival swabs were performed for ocular surface microbiome analysis using conventional culture and next-generation sequencing analysis (NGS). A higher culture-positive rate was found in DM (15%) than in non-DM group (5%) (*p* value = 0.437). Pathogenic organisms and antibiotic-resistant strains were detected in the DR groups (DM-NPDR and DM-PDR). The NGS analysis showed that potentially pathogenic bacteria such as Enterobacteriaceae, Neisseriaceae, *Escherichia-Shigella*, and *Pseudomonas* predominated in DM, especially in DR. There was dissimilarity in the ocular surface microbiome between DM and non-DM groups. The subgroup analysis showed that the DR group had significantly different microbial community from DM-no DR and non-DM groups (*p* value < 0.05). The microbial community in the poorly controlled DM was also significantly different from well-controlled DM and non-DM groups (*p* < 0.001). Using the NGS method, our study is the first to signify the importance of DR and glycemic control status, which affect the changes in the ocular surface microbiome.

## Introduction

Diabetes mellitus (DM), a metabolic disorder characterized by disturbance of glucose metabolism and chronic hyperglycemia, is a significant threat to global health. The prevalence of DM has increased rapidly over the past few decades and is estimated to rise from 463 million in 2019 to 700 million in 2045 worldwide^1^. In Thailand, the estimated number of DM may reach 4.3 million in 2035^2^. The most common eye complication in DM patients is diabetic retinopathy (DR), which is the leading cause of blindness worldwide^2^. Other ophthalmic complications include cataract, glaucoma, and ocular surface changes^3,4^.

The ocular surface changes in DM patients include tear film dysfunction, increased conjunctival metaplasia, decreased conjunctival goblet cell density, decreased sub-basal nerve density, decreased corneal sensitivity, and delayed epithelial and stromal wound healing^5,6^. Previous studies revealed that such changes were more commonly associated with type 2 DM and proportional to the severity of DR and HbA1c level^3,7–9^. Moreover, DM increases the risk of infection such as conjunctivitis, corneal ulcer, and endophthalmitis^10–14^ by altering ocular protective immune response, including decreased cytokine production and impaired cellular immune response functions^15–17^. Alterations of immune function, together with ocular surface changes in DM, may disturb the normal ocular surface microenvironment and microbiome. Moreover, the common treatment modalities in the advanced stage of DR are intravitreal drug injection or intraocular surgery. These procedures may introduce the ocular surface microbes into the eye, resulting in intraocular infection^13,18^. The previous reports showed more bacterial colonization and the presence of gram-negative bacteria on the ocular surface of the DM than in the non-DM groups^19–23^. Consistently, the most common organisms causing postoperative endophthalmitis reported in DM patients were coagulase-negative *Staphylococcus* and gram-negative bacteria, which correlates with conjunctival flora of the DM^13,18,24^.

The culture method is the gold standard for detecting microorganisms. This conventional technique has significant limitations when studying the ocular surface microbiome due to the detection of fastidious microorganisms and small amounts of specimens^25^. Therefore, the development of advanced technology using next-generation sequencing (NGS) has tremendously contributed to the current research. This high-throughput method can generate a million reads per run, thus reducing the cost and analytic time^26–28^. Many studies have reported the changes in the ocular surface microbiome in DM using the culture technique^19–23^, but only a few applied the NGS method^29–32^.

To our knowledge, there is no previous study on the ocular surface microbiome in different stages of DR and the level of glycemic control using the NGS method. Therefore, we conducted a study to identify the difference in the microbial community between DM and non-DM groups using the NGS method and determine the possible effects of the DR severity and glycemic control on the ocular surface microbiome. Results from this study may help improve the understanding of the pathogenesis underlying the increased risks of ocular infections in DM.

## Results

A total of 80 eyes (80 subjects) included 60 eyes of DM and 20 eyes of non-DM subjects. The DM group was further classified into 3 subgroups according to diabetic retinopathy (DR) staging as no diabetic retinopathy (DM-no DR), non-proliferative diabetic retinopathy (DM-NPDR), and proliferative diabetic retinopathy (DM-PDR) (20 eyes/subgroup). The demographics and clinical details are shown in Supplementary Table 1. The mean age of all subjects was 55.6 years old (range 37–79 years old), and 50% were male. Most of the subjects were office workers who resided in the central area of Thailand. The DM subjects were also classified by glycemic control into 2 subgroups, well-controlled DM (HbA1c < 7%) and poorly controlled DM (HbA1c ≥ 7%), which was the majority (60%). The duration of DM ranged from 4 months to 36 years. Among subjects with DR, 13 eyes had diabetic macular edema, and 12 eyes had vitreous hemorrhage.

### Culture and antibiotic susceptibility

A higher culture-positive rate was shown in the DM (15%), compared to the non-DM group (5%), without significant difference (*p* value = 0.437). There were no significant differences in the culture-positive rate among non-DM and the three DM subgroups (*p* value = 0.748). Seven microorganisms were identified, mostly gram-positive cocci, which *Staphylococcus epidermidis* being the most common. In addition, *Providencia rettgeri* was isolated from the DM-NPDR subgroup, while *Kocuria palustris* and *Micrococcus luteus* were isolated from the DM-PDR subgroup. There was no significant difference in the culture-positive rate between well-controlled and poorly controlled DM subgroups (16.7% and 13.9%, respectively, *p* value = 0.571).

Antibiotic-resistant microorganisms were only found in the DM-NPDR and DM-PDR subgroups. Resistance to benzylpenicillin, erythromycin, clindamycin, and fusidic acid in *Staphylococcus hominis* and resistance to ampicillin, cefazolin, and tetracycline in *P. rettgeri* were found in the DM-NPDR subgroup. Methicillin-resistant *S. epidermidis* (MRSE) was isolated from the DM-PDR subgroup. Antibiotic-resistant microorganisms were found in both well-controlled and poorly controlled DM subgroups. The details of the culture and antibiotic susceptibility are shown in Table 1.

**Table 1:** Results of bacterial culture in the non-DM and DM groups (with three subgroups of DR staging).

	Non-DM group	DM group		
		DM-no DR	DM-NPDR	DM-PDR
Growth	1/20 (5%)	3/20 (15%)	3/20 (15%)	3/20 (15%)
**Gram-positive cocci**				
*Staphylococcus epidermidis*	1	1		1^c^
*Staphylococcus aureus*		1		
*Staphylococcus hominis*		1	1^a^	
*Streptococcus mitis*			1	
*Kocuria palustris*				1
*Micrococcus luteus*				1
**Gram-negative bacilli**				
*Providencia rettgeri*			1^b^	

*DM* diabetes mellitus, *Non-DM* non-diabetes mellitus, *No DR* no diabetic retinopathy, *NPDR* non-proliferative diabetic retinopathy, *PDR* proliferative diabetic retinopathy.

^a^Resistant to benzylpenicillin, erythromycin, clindamycin, fusidic acid.

^b^Resistant to ampicillin, cefazolin, tetracycline.

^c^MRSE: methicillin-resistant *Staphylococcus* *epidermidis*.

### Next-generation sequencing analysis

Illumina sequencing of 16S rRNA genes generated 3,698,312 total reads. After quality data processing, 1,786,948 high-quality reads were obtained. There was an average of 22,337 reads per sample (ranging from 4534 to 54,924). The number of amplicon sequence variants (ASVs) was 1359. The approximate saturation of microbial richness of all samples was 4534 sequencing depths, as estimated by the rarefaction curves. The plateau curve in rarefaction was observed when approximately 2000 sequencing depths were reached.

#### Core ocular surface microbiome

The 100% core ASVs sample matching was used to identify the core ocular surface microbiome. At the phylum level, Proteobacteria, Firmicutes, and Actinobacteria were the core microbiome of the non-DM group, and Proteobacteria and Firmicutes of the DM group. At the class level, Alphaproteobacteria, Gammaproteobacteria, Actinobacteria, and Bacilli were the core microbiome of the non-DM group, and Gammaproteobacteria and Bacilli of the DM group.

#### Taxonomic composition of ocular surface microbial community

**Figure 1:**
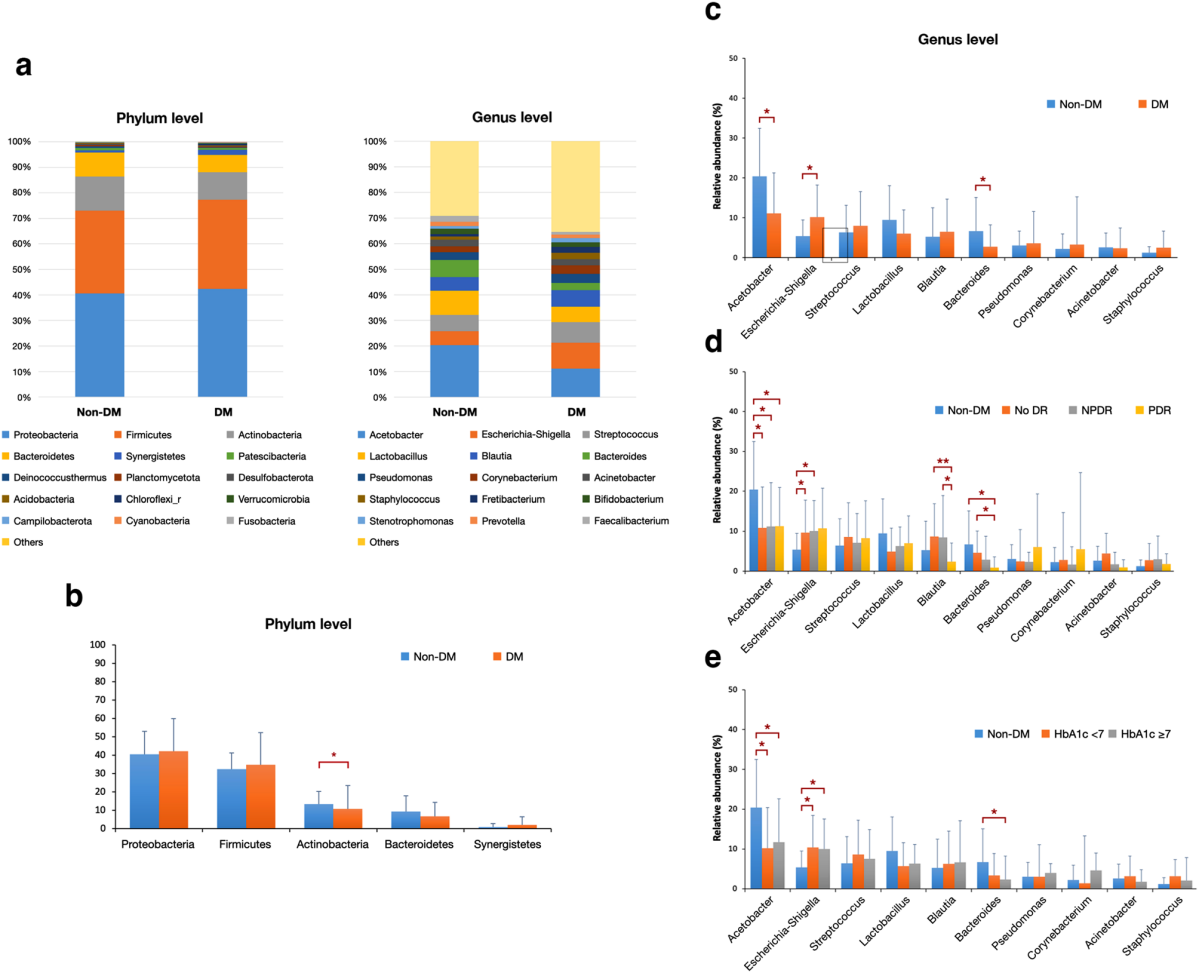
Taxonomic composition (**a**) Top 15 bacteria in phylum and genus levels of the ocular surface microbiome in the non-DM and DM groups. (**b**) Comparison of top 5 phyla between the non-DM and DM groups. (**c**) Comparison of top 10 genera between the non-DM and DM groups, (**d**) between the non-DM and DM subgroups classified by DR staging, and (**e**) between the non-DM and DM subgroups classified by glycemic control. *p value < 0.05; **p value < 0.001. (Abbreviations: *DM* diabetes mellitus, *Non-DM* non-diabetes mellitus, *No DR*  no diabetic retinopathy, *NPDR* non-proliferative diabetic retinopathy, *PDR*  proliferative diabetic retinopathy, *HbA1c* hemoglobin A1c).

Twenty-three bacteria phyla, 40 classes, 190 families, and 318 genera were identified. The bacteria of phylum Proteobacteria were highly abundant in both non-DM and DM groups (40.56% and 42.18%), followed by Firmicutes (32.43% and 34.83%), Actinobacteria (13.43% and 10.71%), and Bacteroidetes (9.35% and 6.71%) (Fig. 1a). The abundance of Actinobacteria was significantly decreased in the DM compared to the non-DM group (*p* value = 0.021) (Fig. 1b). Bacteria in the class of Alphaproteobacteria were significantly abundant in the non-DM group (*p* value = 0.007), while Gammaproteobacteria was significantly abundant in the DM group (*p* value = 0.036). At the family level, Enterobacteriaceae was significantly more abundant in the DM than non-DM groups (*p* value = 0.008). Neisseriaceae was significantly abundant in the DM-NPDR compared to the DM-no DR and non-DM groups (*p* value = 0.016 and 0.029, respectively). At the genus level, *Escherichia-Shigella* was significantly abundant in the DM compared to the non-DM group (*p* value = 0.016) (Fig. 1c). *Pseudomonas* was more abundant in the DM-PDR (6.05%) compared to the DM-NPDR (2.35%), DM-no DR (2.42%), and non-DM group (3.05%), with no statistical significance (*p* value = 0.78) (Fig. 1d). In subgroups classified by glycemic control, *Escherichia-Shigella* was also significantly abundant in both well-controlled (10.38%) and poorly controlled DM (10.02%), compared to the non-DM group (5.39%) (*p* value = 0.028 and 0.035, respectively) (Fig. 1e).

**Figure 2:**
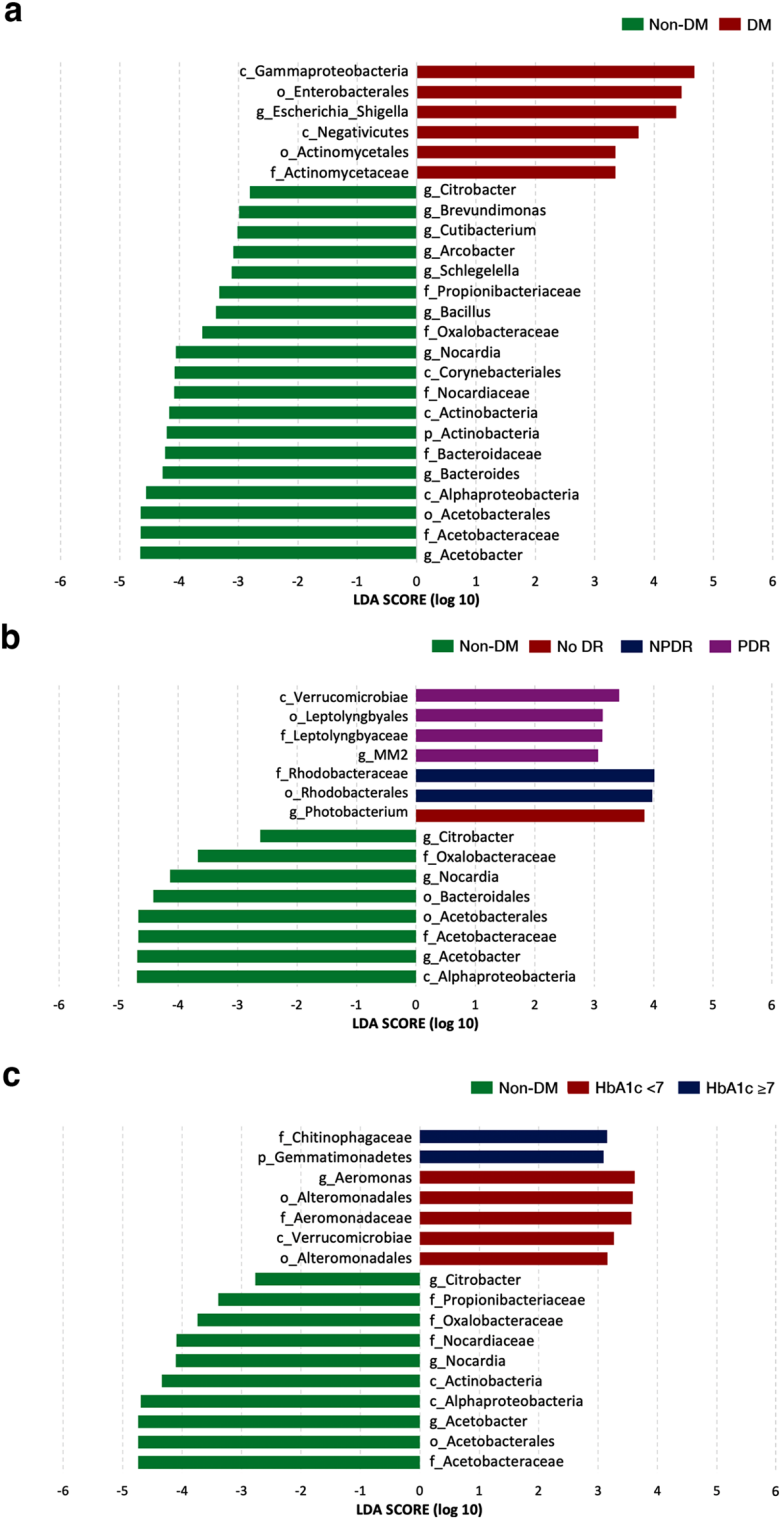
Linear discriminant analysis effect size (LEfSe) represents the significant difference in bacterial distribution between groups. Bacterial taxa with LDA scores greater than 2 were considered significant. (**a**) Comparison of taxa lists between the non-DM and DM groups. (**b**) Comparison of taxa lists between the non-DM and DM subgroups classified by DR staging. (**c**) Comparison of taxa lists between the non-DM and DM subgroups classified by glycemic control. (Abbreviations: *p* phylum, *c* class, *o* order, *f* family, and *g* genus, *DM* diabetes mellitus, *Non-DM* non-diabetes mellitus, *No DR* no diabetic retinopathy, *NPDR* non-proliferative diabetic retinopathy, *PDR* proliferative diabetic retinopathy, *HbA1c* hemoglobin A1c).

To identify the possible biomarkers for differentiating the DM from non-DM subjects, the linear discriminant analysis effect size (LEfSe) was performed to determine the significant difference in the bacterial distribution between groups. The bar plot represents the effect size LDA (LDA; the linear discriminant analysis) for a significant taxon in a particular group. Bacterial taxa with LDA scores greater than 2 were considered significant. The results are shown in Fig. 2.

#### Alpha-diversity

Alpha-diversity was performed using observed ASVs, Chao1, Shannon, and phylogenetic diversity (PD) whole tree. All analyses showed no significant difference between the non-DM and DM groups (*p* value > 0.05). For the subgroups of DM classified by DR staging and glycemic control, there was no statistically significant difference in the alpha-diversity indices between each subgroup. The box plots of the Shannon diversity index are demonstrated in Supplementary Figure 1.

#### Beta-diversity

**Figure 3:**
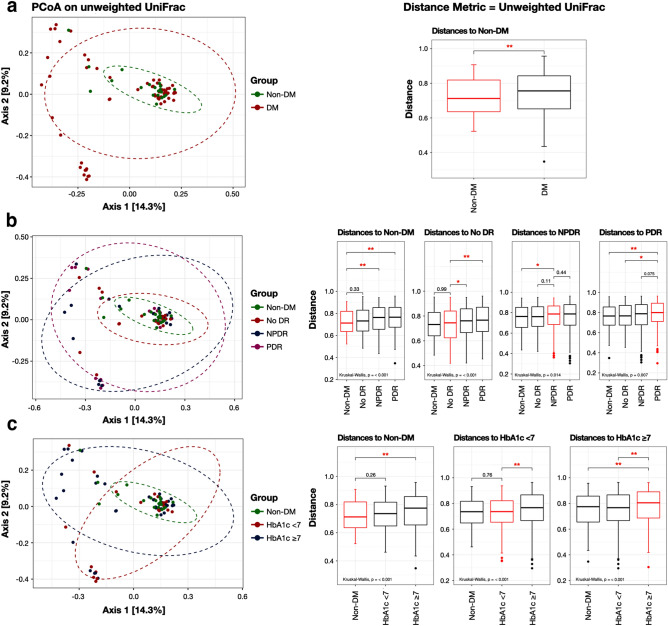
This figure represents beta-diversity between groups by principal coordinate analysis (PCoA) plot based on unweighted UniFrac distance and comparison of ocular surface microbiome between groups (box plot): (**a**) between the non-DM (green-dotted circle) and DM groups (red-dotted circle); (**b**) between the non-DM (green-dotted circle) and DM subgroups classified by the DR staging (no DR: red-dotted circle, NPDR: blue-dotted circle, and PDR: purple-dotted circle); and (**c**) between the non-DM (green-dotted circle) and DM subgroups classified by the glycemic control (HbA1c < 7%: red-dotted circle, HbA1c ≥ 7%: blue-dotted circle) (*p value < 0.05; **p value < 0.001). (Abbreviations: *DM* diabetes mellitus, *Non-DM* non-diabetes mellitus, *No DR* no diabetic retinopathy, *NPDR* non-proliferative diabetic retinopathy, *PDR* proliferative diabetic retinopathy, *HbA1c* hemoglobin A1c).

The principal coordinate analysis (PCoA) using unweighted UniFrac distance showed that the human ocular surface microbial community in the DM group was significantly different from the non-DM group (*p* value = 0.0038) (Fig. 3a). In addition, subgroup analysis showed that the microbial community in the DR subgroup (DM-NPDR and DM-PDR) significantly differed from DM-no DR and the non-DM group (*p* value < 0.05). However, there was no significant difference in the microbial community within the DM-NPDR and DM-PDR subgroups, as well as in the DM-no DR and non-DM groups (both *p* value > 0.05) (Fig. 3b). Interestingly, when classified by glycemic control, the poorly controlled DM had a significantly different microbial community compared to the well-controlled DM and non-DM groups (*p* value < 0.001) (Fig. 3c).

## Discussion

This study is the first to report the ocular surface microbiome in DM according to DR staging and glycemic control using the NGS method. Our results signify the effect of these factors on the ocular surface microbial community. Previous studies described the ocular surface microbiome in DM, mainly using conventional culture technique. The culture-positive rate was shown to be higher in DM than non-DM group (21.7–94.13% and 4.3–73.3%, respectively)^19–23^. The result was reinforced by our study, although not statistically significant (15% and 5%, respectively; *p* = 0.437). The lower culture-positive rate in our study may be due to different specimen collection and culture techniques^19–23^.

Most microorganisms identified were gram-positive cocci with mainly coagulase-negative *Staphylococcus.* Gram-negative bacteria were more commonly found in the DM group, consistent with prior reports^19,20^. Furthermore, the colonization of pathogenic bacteria was more detected in the DM than in the non-DM group^20,21^. *P. rettgeri,* which can be found in normal healthy gut but can lead to opportunistic infection, was found in the DM-NPDR group in this study. Koreishi et al.^33^ reported that *P. rettgeri* was a potential microorganism causing ocular infections such as conjunctivitis, keratitis, dacryocystitis, and endophthalmitis in immunocompromised patients, including DM. *Kocuria* spp., gram-positive bacteria found in the DM-PDR group, was reported to cause canaliculitis, dacryocystitis, recurrent conjunctivitis, and severe marginal keratitis with corneal melt^34,35^.

Martins et al.^20^ reported that DM with DR had more culture-positive rate than DM-no DR subjects. However, this study found no significant difference in culture-positive rate when subgroup analysis by either the DR severity or glycemic control was done. Remarkably, this study detected the antibiotic-resistant organisms only in DM with DR group (including *P. rettgeri* and *S. hominis* in the DM-NPDR group and MRSE in the DM-PDR group). One possible explanation for this finding is that DM with DR subjects is likely to require intraocular intervention and, as a consequence, exposed to antibiotics^36^. For instance, several studies reported the emergence of antibiotic-resistant organisms after multiple sessions of intravitreal drug injection using prophylactic antibiotics^36–38^.

The NGS analysis showed that the top four most common phyla in both non-DM and DM groups were Proteobacteria, Firmicutes, Actinobacteria, and Bacteroidetes, which were consistent with previous studies^30–32^. At the class level, we found that Alphaproteobacteria predominated in the non-DM group, and Gammaproteobacteria predominated in the DM group, similar to the reported from Ham et al.^29^ Prior studies have demonstrated the abundance of potentially pathogenic bacteria in the DM group, using NGS technique^29,32^. Our study likewise found a higher proportion of Enterobacteriaceae, Neisseriaceae, *Escherichia-Shigella,* and *Pseudomonas* in the DM group, especially in DM with the DR group.

The ocular surface microbiome was previously reported to be more diverse in alpha-diversity in the DM compared to the non-DM group^29–32^. Nevertheless, our study using 4 indices did not confirm such findings. The dissimilarity may be from the different sample collection techniques, analysis, and populations. However, from the beta-diversity analysis, we can first demonstrate that the presence of DR and poorly controlled DM status can significantly change the microbial community on the ocular surface. Many groups have reported more severity of ocular surface changes in DR and poorly controlled DM^3,7–9^. As the level of advanced glycation end product (AGE) is significantly increased in patients with DM-PDR^39^. This level affects the ocular surface by reducing proliferation, enhancing apoptosis of epithelial cells, and inducing inflammation in the sub-basal nerve plexus^5,6^. Together with the alteration of protective immune response in DM^15–17^, these can lead to ocular surface microbiome dysbiosis and increase the abundance of potential pathogens in DM, especially in the DR subgroup.

The limitations of this study were small sample size and 16S rRNA incapability to detect viruses and fungi, which may be a part of the ocular surface microbiome. In addition, the data collection in a single time point may not represent the whole picture of ocular surface microbiome change with time. Further studies are undoubtedly warranted to understand the cause-and-effect relationship. Additional assessments in the metagenomics profiling, protein expression, and metabolic activity may lead us to a better understanding of ocular surface microbiome change in this significant and common disease.

## Conclusion

This study demonstrated definite differences in the ocular surface microbiome between the DM and non-DM groups, the DM with DR and no DR groups, and finally, poorly controlled and well-controlled DM groups. In addition, a higher prevalence of pathogenic bacteria and antibiotic-resistant strains was more commonly found in DR compared to the other groups. These results may provide further knowledge on ocular surface diseases and the pathogenesis of many infectious diseases related to the ocular surface microbiome in DM patients.

## Methods

The study was conducted in accordance with the tenets of the Declaration of Helsinki and was approved by the Institutional Review Board, Faculty of Medicine, Chulalongkorn University (IRB No. 377/64 and COA No.704/2021). In addition, the study was registered to the Thai Clinical Trial Registration (TCTR No. TCTR20210427010).

### Subjects and study design

This cross-sectional study was conducted at King Chulalongkorn Memorial Hospital (KCMH), Bangkok, Thailand. The inclusion criteria were subjects aged over 18 years old with or without diabetes mellitus. Exclusion criteria were any subjects with the following conditions: having ocular surface diseases; history of ocular and periocular infection/inflammation; having allergic conjunctivitis; previous contact lens used; history of ocular medication, systemic antibiotic, steroid, or immunosuppressive drug use; history of intravitreal drug injection or ocular surgery within the past three months. This study also excluded the subject, who was a healthcare worker.

A sample size of 20 eyes per group was calculated based on a formula comparing two independent means and standard deviation from the previous study^30^. From February to March 2022, 20 eyes of 20 non-diabetic subjects (non-DM group) with normal blood sugar or HbA1c tests documented within 1 year and 60 eyes of 60 subjects with a known diagnosis of type 2 diabetes mellitus by ADA criteria^40^ (DM group) (total 80 eyes) were recruited. The DM group was further classified by DR staging^41^ into three subgroups: DM-no DR, DM-NPDR, and DM-PDR groups (20 eyes per group). All groups were age- and gender-matched. This same group was also divided into well-controlled DM (HbA1c < 7%) and poorly controlled DM (HbA1c ≥ 7%). Written informed consent was obtained from all subjects before enrollment.

The subjects were interviewed for the demographic data, the Ocular Surface Disease Index (OSDI) questionnaires, and received complete ophthalmic examinations. The conjunctival swab was done before any examinations that touched the ocular surface and the instillation of any drugs, except for topical anesthesia. The processes of conjunctival swab, culture, DNA extraction, and 16S rRNA sequencing processes were done by the masked investigators.

### Specimen collection

The conjunctival swab was collected from one eligible eye of each subject. If both eyes were eligible, only the right eye was included. One drop of 0.5% Tetracaine Hydrochloride solution (Alcon, USA) was applied to the inferior conjunctival fornix of the selected eye. Three minutes later, the subject’s lower eyelid was pulled down to avoid touching the lid margin or eyelashes, and a sterile cotton swab was swept gently on the inferior conjunctival surface from nasal to temporal 5 times without touching eyelids or eyelashes (rotating the sterile cotton swab each time for 360° of swab collection). Then, the swab was placed in 1 ml phosphate buffer saline (PBS) as a sterile transport medium and sent to the microbiology laboratory. All conjunctival swabs were done by the same investigator. A sterile cotton swab was placed in transport media without swabbing (blank swab) and used as a negative control.

### Culture and antibiotic susceptibility test

The sterile pipette was used to transfer the sample onto a chocolate agar plate (approximately 0.1 ml). The plate was incubated in a CO_2_ incubator at 37 °C, 5% CO_2,_ and checked daily for bacterial growth for a week. Bacterial identification was determined by an automated identification system (VITEK 2 XL) and mass spectrometry microbial identification (VITEK MS, France).

The antibiotic susceptibility test was determined using the Kirby-Bauer disk diffusion method and the VITEK 2 XL system. The antibiotics included benzylpenicillin, ampicillin, oxacillin, piperacillin/tazobactam, cefazolin, cefuroxime, cefuroxime axetil, ceftriaxone, cefepime, ertapenem, imipenem, meropenem, amikacin, gentamycin, ofloxacin, ciprofloxacin, levofloxacin, moxifloxacin, erythromycin, clindamycin, chloramphenicol, linezolid, teicoplanin, vancomycin, tetracycline, tigecycline, fusidic acid, rifampicin, and trimethoprim/sulfamethoxazole. The results were interpreted according to the Clinical Laboratory Standards Institute (CLSI) guidelines.

### Next-generation sequencing analysis

Bacterial DNA extraction was performed using the QIAamp DNA Microbiome Kit (QIAGEN, Germany), following the manufacturing protocol. DNA was subjected to 16S metagenomic sequencing library preparation. All samples were processed in a single sequencing run.16S rRNA gene was amplified using 341F and 805R primers, targeting V3-V4 variable regions and 2X sparQ HiFi PCR Master Mix (Quantabio, USA). The PCR amplification included an initial denaturation step of 2 min at 98 °C, followed by 30 cycles of 98 °C for 20 s, 55 °C for 30 s, and 72 °C for 1 min, followed by a final extension step at 72 °C for 1 min for one time. Subsequently, 16S amplicons were purified using sparQ Puremag Beads (Quantabio, USA) and indexed using 2.5 µL of each Nextera XT index primer in a 25 µl PCR reaction, followed by 10 cycles of PCR amplification condition above. The final PCR products were cleaned, pooled, and diluted to the final loading concentration at 4 pM. Cluster generation and 250-bp paired-end read sequencing were performed on an Illumina MiSeq (Illumina, USA) at the Omics Sciences and Bioinformatics Center (Chulalongkorn University, Bangkok, Thailand).

The raw sequences were categorized into groups based on the 5’ barcode sequences. The sequences were processed following the DADA2 v1.16.0 pipeline (URL: https://benjjneb.github.io/dada2/). The microbial diversity and community structures using unique amplicon sequence variants (ASVs) were described by the DADA2 pipeline^42^. Microbial taxa were classified from Silva version 138 as a reference database^43^. Alpha-diversity index (Observed ASVs, Chao1 richness, Shannon, and PD whole tree) was computed using DADA2 software. For Beta-diversity, principal coordinate analysis (PCoA) of unweighted UniFrac distances were plotted from Phyloseq data. Linear discriminant analysis effect size (LEfSe) was performed to identify the bacterial biomarkers.

### Statistical analysis

Demographic data were analyzed using descriptive statistics. The Chi-square test was used for comparing categorical data variables between groups. Independent *t* test or Mann–Whitney U test was performed for comparing continuous variables between two groups, while analysis of variance (ANOVA) or Kruskal–Wallis test was used for comparing more than two groups. Pairwise comparison of alpha-diversity (Observed ASVs, Chao1, Shannon, and PD whole tree) was calculated using the Kruskal–Wallis test. Permutational multivariate analysis of variance (PERMANOVA) was performed to evaluate the differences in beta-diversity among groups. Moreover, the Kruskal–Wallis sum-rank test was also used in LEfSe analysis to identify bacterial biomarkers that differed significantly in abundant taxon between sample groups. *p* value less than 0.05 was considered statistically significant.

## Supplementary Information

Supplementary Information.

## Data Availability

The data analyzed from the patient’s clinical parameters and conjunctival swab samples that support the results of this study are available upon reasonable request from the corresponding author V.P.

## References

[1] Saeedi P (2019). Global and regional diabetes prevalence estimates for 2019 and projections for 2030 and 2045: Results from the International Diabetes Federation Diabetes Atlas, 9(th) edition. Diabetes Res. Clin. Pract..

[2] Aguirre, F., Brown, A., Cho, N. H., Dahlquist, G., Dodd, S., Dunning, T. et al. IDF Diabetes Atlas: Sixth edition. *Sixth ed. IDF*, 160 p (2013).

[3] Gao Y (2015). Ocular surface changes in type II diabetic patients with proliferative diabetic retinopathy. Int. J. Ophthalmol..

[4] Vieira-Potter VJ, Karamichos D, Lee DJ (2016). Ocular complications of diabetes and therapeutic approaches. Biomed. Res. Int..

[5] Richdale K, Chao C, Hamilton M (2020). Eye care providers' emerging roles in early detection of diabetes and management of diabetic changes to the ocular surface: A review. BMJ Open Diabetes Res Care.

[6] Shih KC, Lam KS, Tong L (2017). A systematic review on the impact of diabetes mellitus on the ocular surface. Nutr. Diabetes.

[7] Gekka M (2004). Corneal epithelial barrier function in diabetic patients. Cornea.

[8] Chang SW, Hsu HC, Hu FR, Chen MS (1995). Corneal autofluorescence and epithelial barrier function in diabetic patients. Ophthalmic Res.

[9] Yoon KC, Im SK, Seo MS (2004). Changes of tear film and ocular surface in diabetes mellitus. Korean J. Ophthalmol..

[10] Chang YS (2020). Risk of corneal ulcer in patients with diabetes mellitus: A retrospective large-scale cohort study. Sci. Rep..

[11] Ansari AS, de Lusignan S, Hinton W, Munro N, McGovern A (2017). The association between diabetes, level of glycaemic control and eye infection: Cohort database study. Prim. Care Diabetes.

[12] Grzybowski A, Kanclerz P, Huerva V, Ascaso FJ, Tuuminen R (2019). Diabetes and phacoemulsification cataract surgery: Difficulties, risks and potential complications. J. Clin. Med..

[13] Phillips WB, Tasman WS (1994). Postoperative endophthalmitis in association with diabetes mellitus. Ophthalmology.

[14] Dev S (1999). Progression of diabetic retinopathy after endophthalmitis. Ophthalmology.

[15] Berbudi A, Rahmadika N, Tjahjadi AI, Ruslami R (2020). Type II diabetes and its impact on the immune system. Curr. Diabetes Rev..

[16] Moutschen MP, Scheen AJ, Lefebvre PJ (1992). Impaired immune responses in diabetes mellitus: Analysis of the factors and mechanisms involved. Relevance to the increased susceptibility of diabetic patients to specific infections. Diabete Metab..

[17] Daoud AK, Tayyar MA, Fouda IM, Harfeil NA (2009). Effects of diabetes mellitus vs. in vitro hyperglycemia on select immune cell functions. J. Immunotoxicol..

[18] Fileta JB, Scott IU, Flynn HW (2014). Meta-analysis of infectious endophthalmitis after intravitreal injection of anti-vascular endothelial growth factor agents. OSLI Retina.

[19] Vadodaria B, Ashtamkar S, Maheshgauri R, Motwani D, Sharma A (2020). Analysis of conjunctival flora in diabetic and non-diabetic individuals and their antibiotic sensitivity pattern. IJCEO.

[20] Martins EN (2004). Aerobic bacterial conjunctival flora in diabetic patients. Cornea.

[21] Karimsab D, Razak SK (2013). Study of aerobic bacterial conjunctival flora in patients with diabetes mellitus. Nepal. J. Ophthalmol..

[22] Domngang Noche C, Tchatchouang B, Fotsing Kwetche PR, Kagmeni G, Bella AL (2019). Ocular bacterial flora and antimicrobial susceptibility profile of a diabetic population in Cameroon: An analytical study. Int. J. Biol. Chem. Sci..

[23] Muralidhar CA, Shaik Khaja M, Anandi V (2019). Significance of normal conjunctival flora in diabetic versus healthy individuals. Trop. J. Ophthalmol. Otolaryngol..

[24] Johnson MW (1997). The endophthalmitis vitrectomy study. Ophthalmology.

[25] Willcox MD (2013). Characterization of the normal microbiota of the ocular surface. Exp. Eye Res..

[26] Mitreva, M. The microbiome in infectious disease. In: *Infectious Diseases* Ch. VIII, 68–74.e62 (2017).

[27] Punetha J, Hoffman EP (2013). Short read (next-generation) sequencing: A tutorial with cardiomyopathy diagnostics as an exemplar. Circ. Cardiovasc. Genet..

[28] Mansi Verma, S. K. & Ayush Puri. Genome sequencing. *Bioinformatics Volume I**: **Data, Sequence Analysis, and Evolution Second Edition* (2017).

[29] Ham B (2018). Distribution and diversity of ocular microbial communities in diabetic patients compared with healthy subjects. Curr. Eye Res..

[30] Li S (2019). How ocular surface microbiota debuts in type II diabetes mellitus. Front. Cell Infect. Microbiol..

[31] Zhang Z (2021). Ocular surface microbiota in diabetic patients with dry eye disease. Investig. Ophthalmol. Vis. Sci..

[32] Zhu X (2021). Conjunctival microbiota in patients with type II diabetes mellitus and influences of perioperative use of topical levofloxacin in ocular surgery. Front. Med. (Lausanne).

[33] Koreishi AF, Schechter BA, Karp CL (2006). Ocular infections caused by Providencia rettgeri. Ophthalmology.

[34] Videkar AK, Pranathi B, Madhuri G, Nooreen N (2019). Kocuria varians—an emerging cause of ocular infections. JMSR.

[35] Mattern RM, Ding J (2014). Keratitis with *Kocuria palustris* and *Rothia mucilaginosa* in vitamin A deficiency. Case Rep. Ophthalmol..

[36] Kaldirim H, Yazgan S, Kirgiz A, Ozdemir B, Yilmaz A (2020). Effect of topical antibiotic prophylaxis on conjunctival flora and antibiotic resistance following intravitreal injections in patients with type II diabetes. KJO.

[37] Sierpina DI, Ng CCT, Rauser ME, Fan JT (2019). Ciprofloxacin and conjunctival flora resistance after intravitreal injection. Ophthalmol. Retina.

[38] Kim SJ, Toma HS (2011). Antimicrobial resistance and ophthalmic antibiotics: 1-year results of a longitudinal controlled study of patients undergoing intravitreal injections. Arch. Ophthalmol..

[39] Sato E (2001). Corneal advanced glycation end products increase in patients with proliferative diabetic retinopathy. Diabetes Care.

[40] American Diabetes Association (2020). Classification and diagnosis of diabetes: Standards of medical care in diabetes-2020. Diabetes Care.

[41] Wilkinson CP (2003). Proposed international clinical diabetic retinopathy and diabetic macular edema disease severity scales. Ophthalmology.

[42] Callahan BJ (2016). DADA2: High-resolution sample inference from Illumina amplicon data. Nat. Methods.

[43] Quast C (2013). The SILVA ribosomal RNA gene database project: Improved data processing and web-based tools. Nucleic Acids Res..

