# Supplementary material for: Ocular surface microbiome in diabetes mellitus
Source: Sci Rep. 2022 Dec 13;12:21527. doi: 10.1038/s41598-022-25722-0 (PMC9747965; doi:10.1038/s41598-022-25722-0)

Ocular Surface Microbiome in Diabetes Mellitus

Orathai Suwajanakorn[1,2], Vilavun Puangsricharern[1,2*], Thanachaporn Kittipibul[1,2], & Tanittha Chatsuwan[3,4]

1 Cornea and Refractive Surgery Unit, Department of Ophthalmology, Faculty of Medicine, Chulalongkorn University, Bangkok, Thailand.

2 Excellence Center of Cornea and Limbal Stem Cell Transplantation, Department of Ophthalmology, King Chulalongkorn Memorial Hospital, Bangkok, Thailand.

3 Department of Microbiology, Faculty of Medicine, Chulalongkorn University, Bangkok, Thailand.

4 Center of Excellence in Antimicrobial Resistance and Stewardship, Faculty of Medicine, Chulalongkorn University, Bangkok, Thailand.

*Email: vilavun@hotmail.com

Supplementary Table 1 Demographic data and baseline clinical characteristics of the DM and non-DM groups

		Non-DM group N=20	DM group				p-value
			Total DM N=60	DM-no DR N=20	DM-NPDR N=20	DM-PDR N=20	
Age (yrs), mean (SD)		55.75 (9.54)	55.55 (8.93)	56.10 (8.81)	55.70 (9.15)	54.85 (9.24)	NA
Gender	Male	10 (50%)	30 (50%)	10 (50%)	10 (50%)	10 (50%)	NA
Laterality	RE	8 (40%)	32 (53.33%)	13 (65%)	7 (35%)	12 (60%)	0.3†
Geographic location							
	Central	18 (90%)	43 (71.67%)	17 (85%)	15 (75%)	11 (55%)	0.52†
	East	1 (5%)	10 (16.67%)	1 (5%)	4 (20%)	5 (25%)	
	Others	1 (5%)	7 (11.67%)	2 (10%)	1 (5%)	4 (20%)	
BMI, mean (SD)		23.45 (3.15)	26.87 (4.25)	28.84 (5.20)	25.66 (3.26)	26.11 (3.49)	0.001†
BCVA logMAR, mean (SD)		0.08 (0.11)	0.45 (0.63)	0.10 (0.13)	0.31 (0.20)	0.93 (0.87)	0.011†
IOP, mean (SD)		15.00 (2.53)	15.17 (3.09)	16.55 (2.42)	14.95 (3.68)	14.00 (2.62)	0.83†
OSDI score, mean (SD)		1.70 (1.59)	1.45 (1.86)	0.89 (1.62)	1.89 (2.09)	1.57 (1.78)	0.6†
TBUT, mean (SD)		11.80 (1.24)	11.30 (1.52)	11.75 (1.68)	11.30 (1.45)	10.85 (1.35)	0.19†
Duration of DM (yrs), median (IQR)		NA	7 (5, 13)	7 (6, 10)	10 (7, 16)	5 (3, 19)	0.13††
FBS, mean (SD)		91.7 (5.13)	145.52 (52.58)	124.5 (24.26)	149.3 (65.54)	162.75 (53.92)	<0.001† 0.063 ††
HbA1c (%), mean (SD)		5.06 (0.28)	7.69 (1.92)	6.99 (0.67)	8.02 (2.58)	8.07 (1.87)	<0.001† 0.13††
HbA1c control (n)							
< 7 %: Well-controlled DM		NA	24 (40%)	9 (45%)	8 (40%)	7 (35%)	0.81††
≥ 7 %: Poorly controlled DM		NA	36 (60%)	11 (55%)	12 (60%)	13 (65%)	
Diabetic macular edema		NA	13 (21.67%)	NA	6 (30%)	7 (35%)	NA
Vitreous hemorrhage		NA	12 (20%)	NA	NA	12 (60%)	NA

† : p-value comparison between non-DM and DM group

††: p-value comparison between subgroups classified by DR staging

(Abbreviations: DM=diabetes mellitus, Non-DM=non-diabetes mellitus, No DR= no diabetic retinopathy, NPDR=non-proliferative diabetic retinopathy, PDR=proliferative diabetic retinopathy, BMI=body mass index, BCVA=best corrected visual acuity, IOP=intraocular pressure, OSDI=ocular surface disease index, TBUT=tear film break up time, FBS=fasting blood sugar, HbA1c=hemoglobin A1c, NA=not available)

Supplementary Figure 1 Shannon diversity indices show no significant difference in alpha-diversity between groups (p-value >0.05) as follows: (a) between the non-DM and DM groups; (b) between the non-DM and DM subgroups classified by DR staging; and (c) between the non-DM and DM subgroups classified by glycemic control.

(Abbreviations: DM=diabetes mellitus, Non-DM=non-diabetes mellitus, No DR= no diabetic retinopathy, NPDR=non-proliferative diabetic retinopathy, PDR=proliferative diabetic retinopathy, HbA1c=hemoglobin A1c)

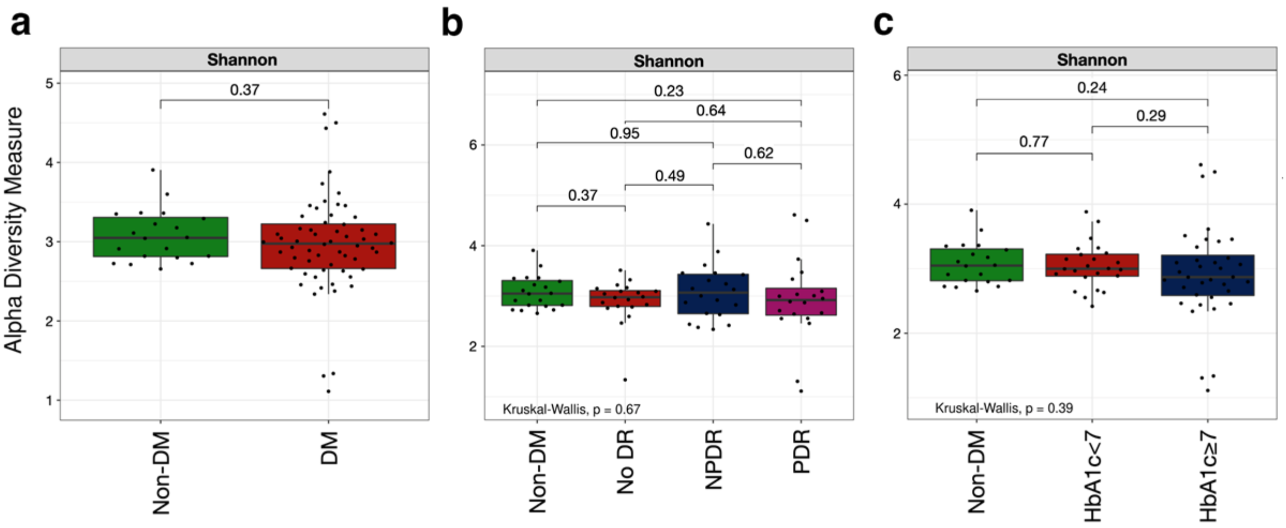

Supplement: Supplementary file 1 — Supplementary Information. [file 41598_2022_25722_MOESM1_ESM.pdf]
